# Evaluation of two main RNA-seq approaches for gene quantification in clinical RNA sequencing: polyA+ selection versus rRNA depletion

**DOI:** 10.1038/s41598-018-23226-4

**Published:** 2018-03-19

**Authors:** Shanrong Zhao, Ying Zhang, Ramya Gamini, Baohong Zhang, David von Schack

**Affiliations:** 1 Precision Medicine, Early Clinical Development, Pfizer Worldwide Research and Development, Cambridge, 02139 MA USA; 2 Inflammation & Immunology Research Unit, Pfizer Worldwide Research and Development, Cambridge, MA 02139 USA

## Abstract

To allow efficient transcript/gene detection, highly abundant ribosomal RNAs (rRNA) are generally removed from total RNA either by positive polyA+ selection or by rRNA depletion (negative selection) before sequencing. Comparisons between the two methods have been carried out by various groups, but the assessments have relied largely on non-clinical samples. In this study, we evaluated these two RNA sequencing approaches using human blood and colon tissue samples. Our analyses showed that rRNA depletion captured more unique transcriptome features, whereas polyA+ selection outperformed rRNA depletion with higher exonic coverage and better accuracy of gene quantification. For blood- and colon-derived RNAs, we found that 220% and 50% more reads, respectively, would have to be sequenced to achieve the same level of exonic coverage in the rRNA depletion method compared with the polyA+ selection method. Therefore, in most cases we strongly recommend polyA+ selection over rRNA depletion for gene quantification in clinical RNA sequencing. Our evaluation revealed that a small number of lncRNAs and small RNAs made up a large fraction of the reads in the rRNA depletion RNA sequencing data. Thus, we recommend that these RNAs are specifically depleted to improve the sequencing depth of the remaining RNAs.

## Introduction

RNA sequencing (RNA-seq) has revolutionized the way biologists examine transcriptomes and has been successfully applied in biological research, drug discovery, and clinical development^1–3^. Compared with microarray-based transcriptome profiling, RNA-seq has a wider dynamic range and avoids some of the technical limitations such as varying probe performance and cross-hybridization^4^. RNA-seq can measure the expression levels of thousands of genes simultaneously and provide insights into functional pathways and the regulatory networks in biological systems. In addition, RNA-seq can provide novel insights into alternative splicing^5^, unannotated exons, and novel transcripts^6^.

Ribosomal RNA (rRNA) is the most highly abundant component of total RNA isolated from animal or human cells and tissues, comprising the majority (>80% to 90%) of the molecules in a total RNA sample^7^. To allow efficient transcript/gene detection, highly abundant rRNAs must be removed from total RNA before sequencing. Standard approaches include selection of polyadenylated RNA (polyA) transcripts using oligo (dT) primers, and depletion of highly abundant rRNAs through hybridization capture followed by magnetic bead separation. However, the polyA+ selection and rRNA depletion methods each have unique advantages and limitations. polyA+ selection is used for most transcriptome studies because the sequencing depth required is relatively low when the focus is mainly on the protein-coding fraction of a transcriptome^8^. Several recent studies have noted that although rRNA-depleted RNA libraries cost more than polyA+ selection libraries to achieve comparable coverage of protein-coding reads in a transcriptome, it provides more information on polyA- transcripts. Another technical advantage that favours RNA-seq of rRNA-depleted libraries compared with polyA-selected libraries is that its performance is better for degraded RNAs^8–12^.

Since 2010, comparisons between polyA+ selection and rRNA depletion methods have been carried out by various groups using different kits, cell lines, and samples^8,10,11,13–16^. However, the assessments of different RNA-seq protocols have relied mostly on animal samples or cell-line-derived RNAs. In this paper, we evaluated the two sequencing protocols in clinical settings where RNA samples were collected primarily from human blood and tissues. Blood samples from individuals were pooled prior to data generation to remove any possible association of analytical measurements with a single donor. Four replicate samples from pooled blood and four replicates from colon tissue were sequenced, using both protocols. Our analyses showed that the rRNA depletion method captured a wider diversity of unique transcriptome features, whereas the polyA+ enrichment method outperformed the rRNA depletion method in exonic coverage and accuracy of gene quantification. In the rRNA depletion method, many more reads were mapped to intronic regions, which not only significantly reduced the number of usable reads for exon/gene quantification but also led to overestimation of the expression levels for the genes that overlapped with the intronic regions of other genes. For the blood and colon samples, 220% and 50% more reads, respectively, had to be sequenced in the rRNA depletion method to achieve the same level of exonic coverage as the polyA+ selection approach. Our results show that selection of the library preparation protocol in clinical research should be guided by the study objectives, and polyA+ selection is recommended for RNA-seq projects where the main goal is quantification of protein-coding genes.

## Results

### Experimental design and RNA-seq quality control metrics

**Figure 1:**
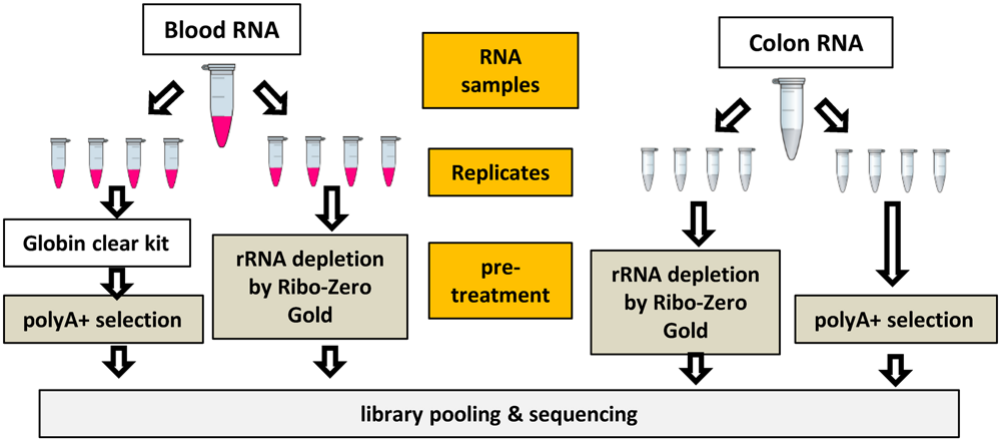
Experimental design used in this study. Four technical replicates per condition were sequenced using both the polyA+ selection and rRNA depletion protocols.

To fairly evaluate the differences between the two protocols and the reproducibility of the RNA-seq data, we minimized the confounding factors as much as possible. The overall experimental design is depicted in Fig. 1. The blood RNA samples were collected from five healthy volunteers and then pooled. The colon sample was from a single donor. Blood was chosen because it is easy to collect and commonly used in clinical RNA-seq studies. Colon tissue was chosen because it is closely related with inflammatory bowel disease and colon cancer^17^. Four technical replicates per condition were sequenced using both the polyA+ selection and rRNA depletion protocols. The rRNA depletion kits used in this study were Ribo-Zero Gold for colon RNA and Globin-Zero for blood (both abbreviated as RiboZ). After sequencing, 50 M reads were randomly sampled from each replicate library, and then processed by an in-house developed QuickRNASeq.^18^ pipeline.

**Figure 2:**
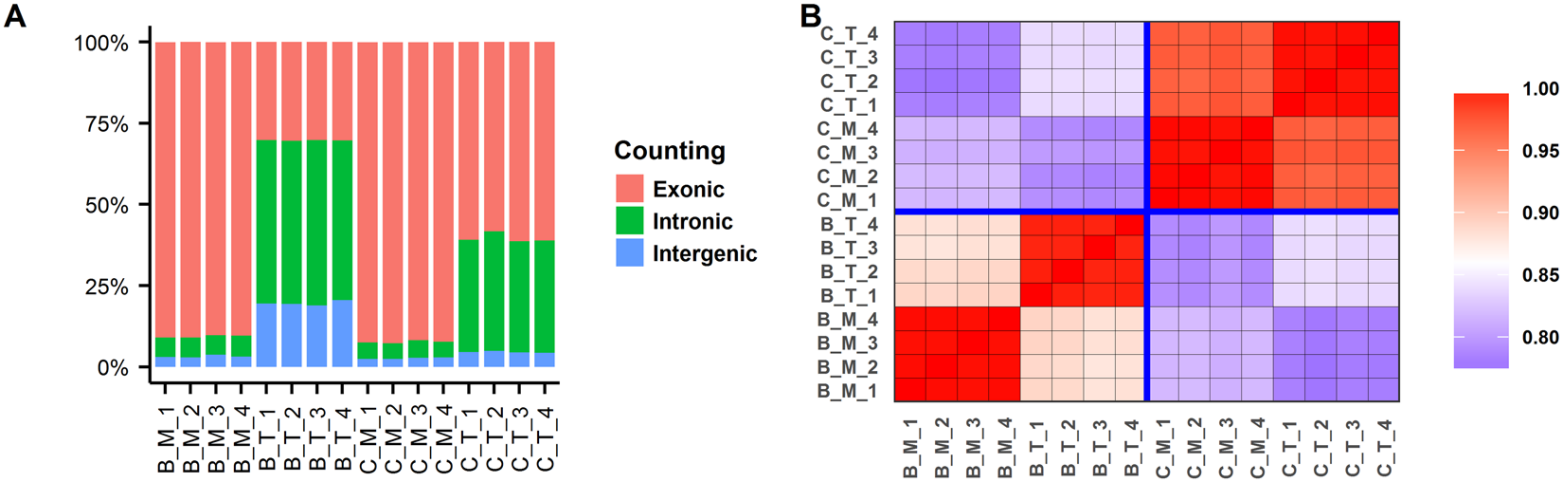
RNA-seq quality control metrics. (**A**) Summary of reads counting; (**B**) Correlation of gene expressions among the samples. First, lowly expressed genes (RPKM < 0.5 across all samples) were filtered out, and then correlation was calculated using log2(CPM + 1). CPM denotes Count Per Million. Replicates are named as X_Y_N, where X indicates tissue (B, blood; C, colon); Y indicates the RNA-seq library preparation protocol (M, polyA+ selection; T, rRNA depletion), and N indicates the replicate number (1 to 4).

The annotations for the blood and colon samples and replicates, the number of reads uniquely mapped to the human reference genome GRCh38, and the number of reads falling in exonic regions in human Gencode Release 25 are summarized in Table 1 and Fig. 2A. For the number of reads mapped to the genome (*Unique_Mapped* in Table 1), there was very little difference between the polyA+ selection and rRNA depletion methods, but for the read counts falling in exonic regions (*Exonic_Reads* in Table 1 and Fig. 2A), the differences were much more pronounced, especially for blood. A very high portion of reads (more than half in blood and one third in colon) mapped to intronic regions in the rRNA depleted libraries (Fig. 2A). The pattern in Fig. 2A indicates many immature and/or nascent RNA transcripts were captured in the rRNA depletion RNA-seq. The correlation of gene expression levels among all samples is shown in Fig. 2B. All the technical replicates were clustered together and arrayed along the diagonal line, and clearly were very highly correlated. However, as expected, a significant difference was observed between blood and colon samples. The scatter plots for blood samples and colon samples are shown in Supplementary Figs S1 and S2, respectively. The concordance between the polyA+ selection and rRNA depletion methods was higher in colon than in blood, indicating more immature RNA transcripts were captured in blood than in colon samples.

**Table 1:** RNA-seq quality control metrics.

Sample	Replicate^a^	Source	Protocol^b^	Total_reads	Unique_Mapped	Exonic_Reads^c^	Exonic_Reads (%)
*Blood_PolyA*	B_M_1	blood	polyA+	50,000,000	43,215,282	38,254,168	76.51
	B_M_2	blood	polyA+	50,000,000	42,476,355	37,646,793	75.29
	B_M_3	blood	polyA+	50,000,000	42,185,029	37,072,203	74.14
	B_M_4	blood	polyA+	50,000,000	42,954,993	37,813,280	75.63
*Blood_RiboZ*	B_T_1	blood	RiboZ	50,000,000	41,513,424	11,665,272	23.33
	B_T_2	blood	RiboZ	50,000,000	41,614,111	11,714,372	23.43
	B_T_3	blood	RiboZ	50,000,000	41,637,819	11,641,934	23.28
	B_T_4	blood	RiboZ	50,000,000	41,265,342	11,405,741	22.81
*Colon_PolyA*	C_M_1	colon	polyA+	50,000,000	43,281,953	38,971,070	77.94
	C_M_2	colon	polyA+	50,000,000	44,375,310	40,053,155	80.11
	C_M_3	colon	polyA+	50,000,000	42,908,688	38,368,949	76.74
	C_M_4	colon	polyA+	50,000,000	44,432,717	39,891,693	79.78
*Colon_RiboZ*	C_T_1	colon	RiboZ	50,000,000	42,294,547	23,989,467	47.98
	C_T_2	colon	RiboZ	50,000,000	41,719,947	22,908,423	45.82
	C_T_3	colon	RiboZ	50,000,000	42,491,691	24,288,251	48.58
	C_T_4	colon	RiboZ	50,000,000	42,028,918	23,779,962	47.56

^a^Replicates are named as X_Y_N, where X indicates tissue (B, blood; C, colon); Y indicates the RNA-seq library preparation protocol (M, polyA+ selection; T, rRNA depletion), and N indicates the replicate number (1 to 4).

^b^PolyA+ indicates polyA+ selection; RiboZ indicates rRNA depletion by Ribo-Zero Gold (for colon) or Globin-Zero (for blood).

^c^A read was considered exonic if it overlapped with an exon of any annotated gene. The gene model is human Gencode Release 25.

### Comparison of detected genes across protocols and breakdown of sequences by gene biotype.

The read counts for individual genes across replicates of the same group (Table 1) were merged, and then the numbers of expressed genes were compared between the polyA+ selection and rRNA depletion protocols. After merging of replicates, four combinations of samples and protocols were obtained, i.e. *Blood_PolyA*, *Blood_RiboZ*, *Colon_PolyA*, and *Colon_RiboZ*.

**Figure 3:**
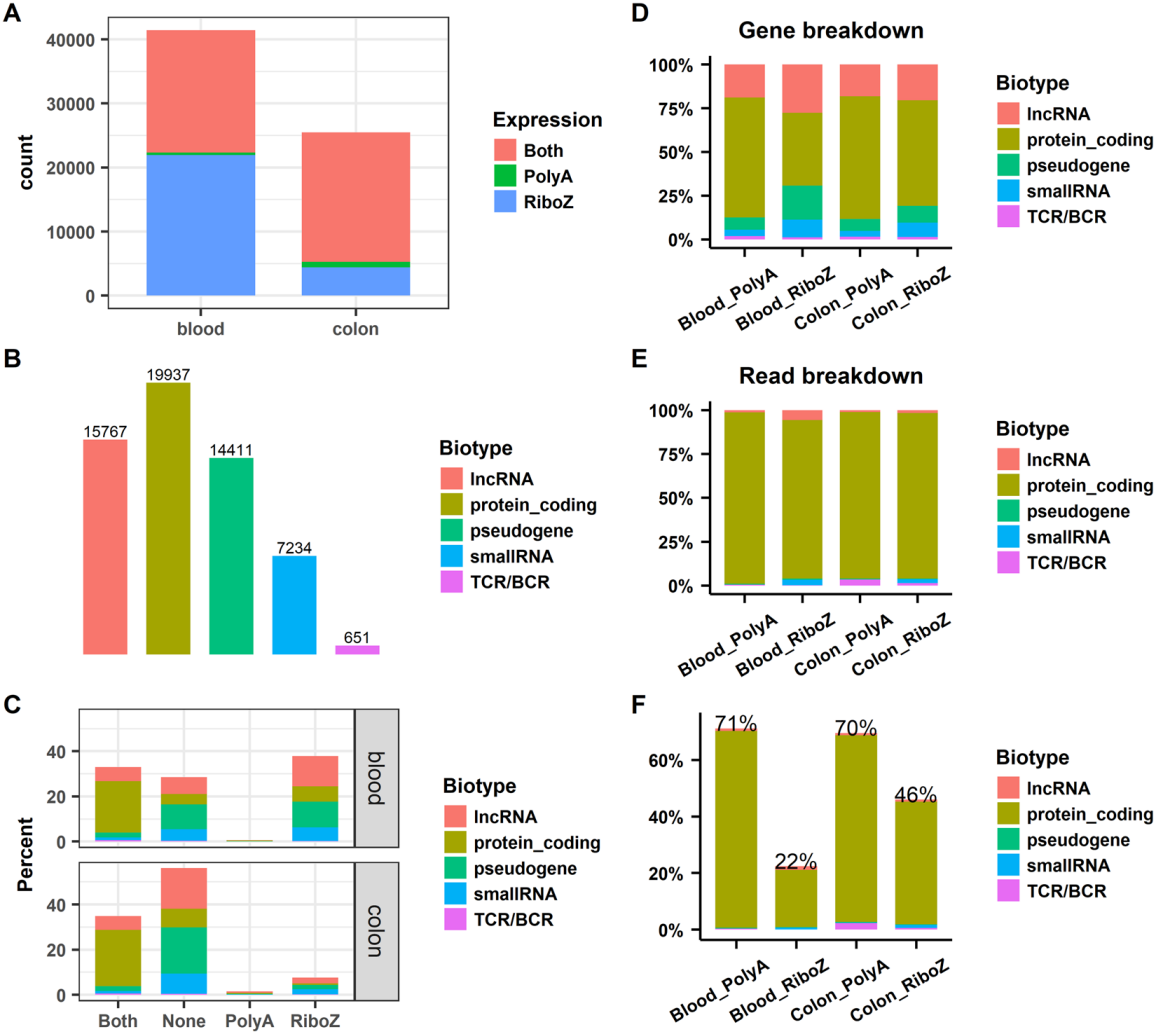
Comparison of detected genes across protocols and breakdown of sequences by gene biotype. (**A**) Number of expressed genes in the blood and colon samples; (**B**) Classification of the 58,000 annotated genes in Gencode Release 25 by biotype; (**C**) Split of the expressed genes in panel 3 A by the gene biotypes in panel 3B; (**D**) Fraction of expressed genes in each biotype in the individual samples; (**E**) Fraction of total counted reads in each biotype in the individual samples; and (**F**) Fraction of usable reads for gene quantification normalized based on the corresponding library size. *Both*, genes detected by both protocols; *PolyA*, genes detected only by polyA+ selection; *RiboZ*, genes detected only by rRNA depletion; *Blood_PolyA* and *Blood_RiboZ*, blood samples processed by polyA+ selection and rRNA depletion, respectively; *Colon_PolyA* and *Colon_RiboZ*, colon samples processed by polyA+ selection and rRNA depletion, respectively.

A gene was considered as expressed if its RPKM (reads per kilobase of transcript per million mapped reads) in a sample was >0.1^19^. Accordingly, all annotated genes in human Gencode Release 25^20^ were divided into four groups according to their expression levels in the two protocols, namely, Both (detected by both protocols), PolyA (detected only by polyA+ selection), RiboZ (detected only by rRNA depletion), and None (detected by neither protocol). The numbers of detected genes in each group in blood and colon are shown in Fig. 3A. While many genes were detected by both protocols, some genes were detected only by polyA+ selection or rRNA depletion. The polyA+ selection protocol should almost exclusively detect the transcripts with polyA+ tails, whereas the rRNA depletion protocol should capture both polyA+ and polyA− transcripts. Therefore, genes detected by polyA+ selection, in principle, should also be detected by rRNA depletion, but the reverse should not be true. Fig. 3A shows that very few genes were detected only by polyA+ selection, whereas many more genes were detected only by rRNA depletion, especially in blood.

All the genes in Gencode Release 25 can be classified into five biotype categories: protein-coding, lncRNA (long noncoding RNA), pseudogene, small RNA, and TCRs and BCRs (T- and B-cell receptors). The number of genes in each category is shown in Fig. 3B. Accordingly, the expressed genes, shown in Fig. 3A, can be split according to their biotypes, and the bar plot in Fig. 3C shows their corresponding percentages. For colon, genes detected only by rRNA depletion were primarily lncRNAs, pseudogenes, and small RNAs. For blood, additional protein-coding genes, lncRNAs, pseudogenes, and small RNAs were detected only by rRNA depletion.

Next, we split the expressed genes and the total counted reads in individual samples by gene biotype (Fig. 3D and E), and found that the observed patterns for the genes and reads were very different. For example, for *Blood_PolyA* in Fig. 3D, 68% of the expressed genes were protein-coding genes, followed by lncRNAs (19%), pseudogenes (7%), small RNAs (4%), and TCRs/BCRs (<2%). Whereas, for *Blood_PolyA* in Fig. 3E, the sequence reads were predominantly protein-coding genes (>98%), with all other categories collectively representing < 2% of all the reads. The comparisons between expressed genes and counted reads (Fig. 3D and E) indicate the expression of protein-coding genes is, on average, much higher than the expression of lncRNAs and pseudogenes.

We observed that a large fraction of reads mapped to intronic regions (Fig. 2A). To fairly compare the number of usable reads for gene quantification, we normalized the observed read counts by the total number of sequenced reads (ratios are shown in Fig. 3F). As expected, the percentages of usable reads in the polyA+ selection method (71% for blood and 70% for colon) were much higher than in the rRNA depletion method (22% for blood and 46% for colon). To reach the same level of exonic read coverage for the colon and blood samples, about 50% and 220% more reads would need to be sequenced using rRNA depletion RNA-seq compared with polyA+ selection RNA-seq. Considering the large differences in usable reads (71% vs 22% in blood and 70% vs 46% in colon) for gene quantification, we recommend using the polyA+ selection protocol if the primary goal of a RNA-seq study is to quantify expression levels of protein-coding genes. Conversely, if the primary goal is to study histone or lncRNAs then a rRNA depletion method could be cautiously considered.

### Concordance of gene expression between polyA+ selection and rRNA depletion RNA-seq

**Figure 4:**
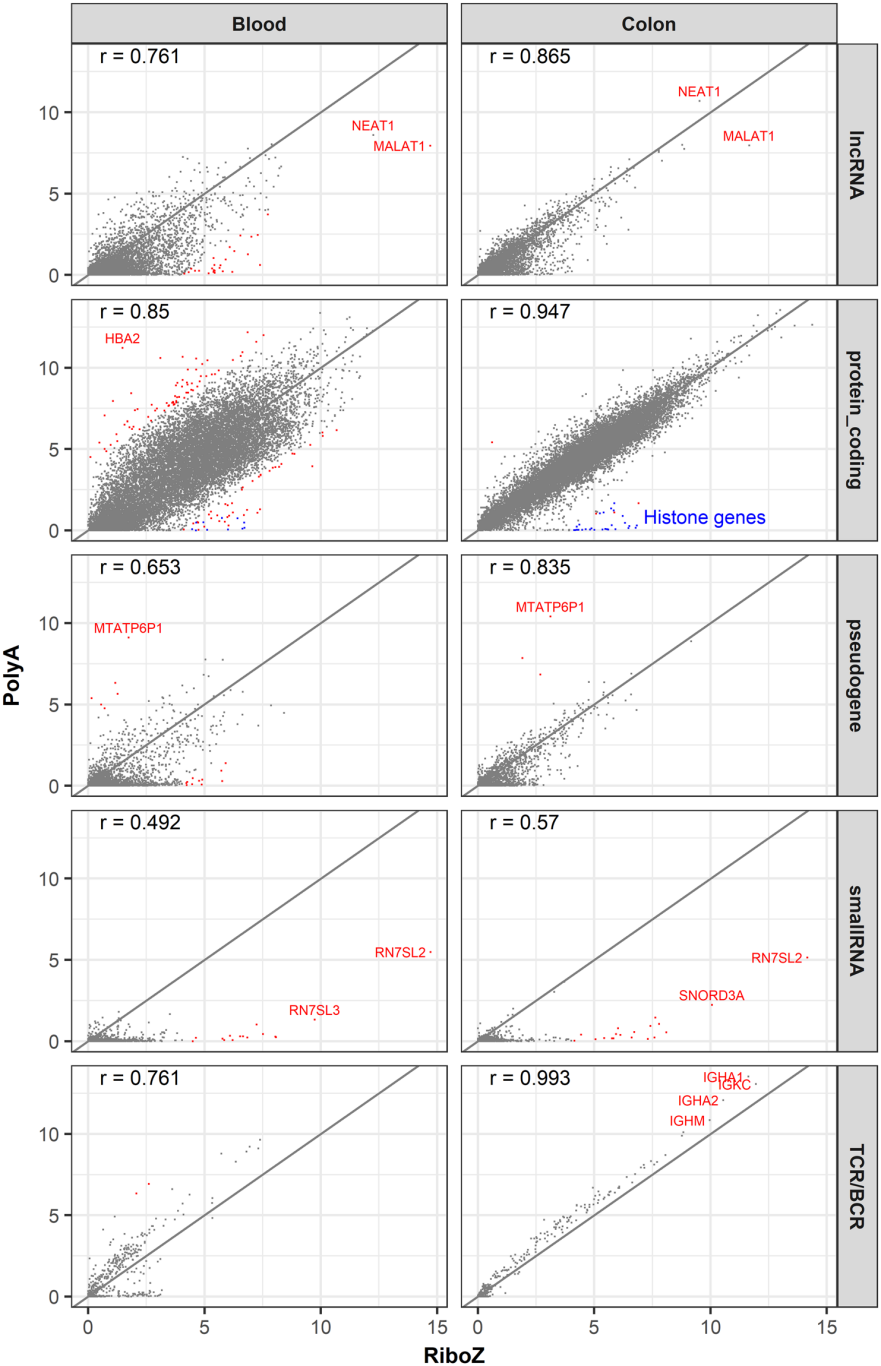
Concordance of gene expression between the polyA+ selection and rRNA depletion RNA-seq data. The correlation coefficients are shown at top-left corner of each plot. The diagonals are shown as solid lines. The x- and y-axes indicate log2(CPM+1). CPM is counts per million. Genes with a log2FC (fold change) >4 are in red. Genes with exceptionally high expressions and large differences between the two protocols are labelled.

The scatter plots of gene expression for blood and colon samples in Fig. 4 are split by gene biotypes. In general, the concordance between the two methods was much higher in colon than in blood, and this was especially true for protein-coding genes. Although the overall concordance was high, it clearly varied from biotype to biotype. Protein-coding genes had the highest concordance, and small RNAs and TCRs/BCRs had the lowest concordance in the two methods. The concordance for lncRNAs and pseudogenes was somewhere in between. When there are large discrepancies between the expressions reported by polyA+ selection and rRNA depletion, which protocol is more trustworthy will be discussed later in the section of “Accuracy in gene quantification between polyA+ selection and rRNA depletion”.

**Figure 5:**
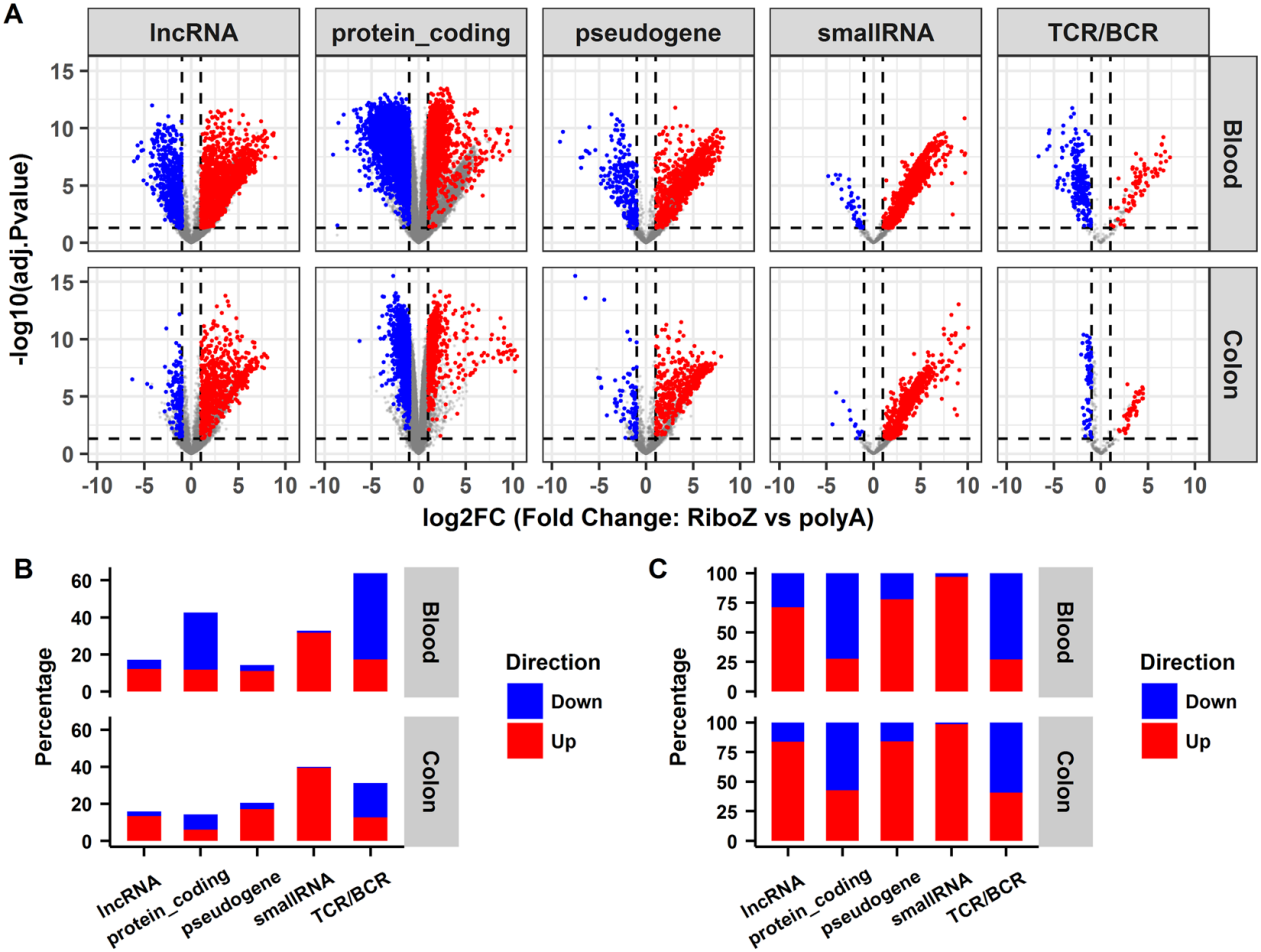
Differential expression analysis between the rRNA depletion and the polyA+ selection RNA-seq data. (**A**) Volcano plots of differentially expressed genes in rRNA depletion compared with poly+ selection. *RiboZ*, rRNA depletion; polyA+, poly+ selection. (**B**) Summary of the percentages of differentially expressed genes in each biotype split by the direction of change. (**C**) Fraction of up- and down-regulated genes normalized by the number of differentially expressed genes in each biotype. Up-regulated genes are in red and down-regulated genes are in blue in the rRNA depletion vs poly + selection comparisons.

Differential gene expression analysis was performed using edgeR^21^ and the results are summarized in Fig. 5. The four replicates per condition were treated as a group. Volcano plots for the differentially expressed genes between the rRNA depletion and polyA+ selection methods for the blood and colon samples are shown in Fig. 5A, separated by gene biotypes. A gene was defined as differentially expressed if (1) its fold change was >2, (2) its Benjamini-Hochberg adjusted p-value was <0.05, and (3) the mean expression was >0.5 RPKM. Figure 5B summarizes the percentages of differentially expressed genes in each category split by direction of change. The percentages were calculated by dividing the number of differentially expressed genes by the total number of expressed genes in each category. In Fig. 5C, the percentages were normalized with respect to the total number of differentially expressed genes, which better reflects the direction of change in each biotype category. Notably, differentially expressed lncRNAs or pseudogenes tended to have higher expressions in the rRNA depletion compared with the polyA+ selection method. This is because either such transcripts were captured only by rRNA depletion, or were overestimated due to overlapping with intron regions of other genes. It is quite unusual that nearly all the differentially expressed small RNAs had higher expressions in the rRNA depletion method. By in-depth investigation, we discovered the high expressions for many of the small RNA genes in the rRNA depletion RNA-seq were false positives, and this will be discussed in detail in the next section.

### Accuracy in gene quantification between polyA+ selection and rRNA depletion RNA-seq

Despite the overall high concordance between the two protocols, large discrepancies were observed for thousands of genes (Figs 4 and 5). We therefore carefully investigated the extreme cases noted in Fig. 4, and randomly chose some of the differentially expressed small RNAs and TCRs/BCRs in Fig. 5 to illustrate the potential drawbacks of the rRNA depletion method for gene quantification.

*MALAT1* (metastasis-associated lung adenocarcinoma transcript 1) is broadly expressed and is among the most abundant lncRNAs in mouse and human tissues^22^. The dominant isoform is unspliced and expressed at levels similar to or higher than many protein-coding house-keeping genes, including β-actin and *GAPDH*^23^. *MALAT1* alone contributed about 2.7% of the total counted reads (and >47% of the reads mapped to lncRNAs, Supplementary Table S1) in *Blood_RiboZ*. The majority of *MALAT1* transcripts with polyA+ tails are processed by RNase P cleavage to generate mature transcripts with their 3′ termini protected by a triple helical structure^24^. Almost always, the cleavage occurred several hundred nucleotides upstream of the polyA+ signal. Because transcripts with polyA+ signals represent only a tiny fraction of all *MALAT1* transcripts, *MALAT1* expression was much lower in the polyA+ selection than in the rRNA depletion method. The *NEAT1* (nuclear paraspeckle assembly transcript 1) locus is regulated by alternative 3′-end processing^24^. The primary transcript of *NEAT1* can be cleaved either by the canonical cleavage and polyadenylation machinery to generate a polyadenylated RNA or by the tRNA biogenesis machinery to generate a non-polyadenylated RNA with a mature 3′-end that is protected by a triple helix^24^. *NEAT1* was very highly expressed in both blood and colon, regardless of the sequencing protocols. A recent study demonstrated that high *NEAT1* expression was associated with a poor prognosis in cancer patients^25^.

The observed large differences for *HBA2* (haemoglobin alpha 2) expression in the blood sample likely results from differences in the efficiencies of globin removal of the different kits. Because globin transcripts can account for up to 70% of the total whole blood mRNA population^26^, different globin reduction protocols have been used successfully in gene expression studies^27^ to improve the sensitivity of gene expression profiling experiments. The Globin-Zero Gold rRNA Removal Kit can remove both globin and mitochondrial and cytoplasmic rRNAs. This kit seemed to deplete globin much more effectively than the Globin-Zero Kit, which likely explains why the reported *HBA2* expression was much lower in the rRNA depletion approach than in the polyA+ selection method. We were perplexed by the large expression difference for the pseudogene *MTATP6P1* (mitochondrially encoded ATP synthase 6 pseudogene 1) between the two protocols, but found that the expression pattern of *MTATP6P1* was nearly the same as its functional counterpart *MT-ATP6* (mitochondrially encoded ATP synthase 6) across the different samples (Supplementary Fig. S3). Therefore, we speculated that the Globin-Zero Gold rRNA Removal Kit likely depleted both the *MT-ATP6* and *MTATP6P1* transcripts, and that is why the polyA+ selection method reported high expression of *MTATP6P1*, while the rRNA depletion approach did not.

The expressions of a subset of histone genes are shown in Supplementary Fig. S4. The majority of histone genes are known to be expressed as replication-dependent polyA− transcripts^28,29^, and thus the majority of them are barely detected in the polyA+ selection RNA-seq. However, a small number of histone genes did show appreciable expression in the polyA+ selection method, and *HIST1H2AC* is a case in point (Supplementary Fig. S4). Indeed, the expression of *HIST1H2AC* in *Blood_PolyA* was as high as 151 counts per million. We speculated that *HIST1H2AC* had a polyA+ tail, and the 3′-end sequencing confirmed a polyA+ signature in *HIST1H2AC* (Supplementary Fig. S5). Recently, it has been reported that a subset of histone genes produces polyA+ mRNAs under a variety of cellular conditions^30,31^. Therefore, whether a gene has a polyA+ tail is not simply black and white, and likely to be more complicated and dynamic than was first thought.

The small RNAs, *RN7SL2*, *RN7SL3*, and *SNORD3A*, displayed very high expression levels in the rRNA depletion RNA-seq (Fig. 4, Supplementary Table S1). *SNORD3A* is a known abundant small nucleolar RNA (snoRNA) involved in the processing of rRNA precursors^32^. *RN7SL2* and *RN7SL3* are part of the signal recognition particle complex, which mediates the cotranslational insertion of secretory proteins into the lumen of the endoplasmic reticulum^33^. The exceptionally high expressions of these small RNAs in HEK293 cell line and extracellular vesicles have also been reported^16,34^. The *RN7SL2* reads accounted for about 88% (in blood) and 84% (in colon) of reads derived from small RNAs. Sultan *et al*.^16^ reported exceptionally high expression of *RN7SL1* rather than *RNSL2*. *RN7SL1*, *RN7SL2*, and *RN7SL3* are paralogous genes, and sequence comparison revealed they share high sequence identity.

Notably, a large number of small RNAs were detected only in rRNA depletion compared with polyA+ selection RNA-seq (Fig. 4). The library construction methods used in this study included size selection steps, where smaller fragments were presumably removed. Therefore, the investigation of small non-coding RNAs, including mature micro RNAs (miRNAs), Piwi-interacting RNAs (piRNAs), small nuclear RNA (snRNA), and some snoRNAs will be limited because of the preparation methods used here. To understand why the rRNA depletion RNA-seq detected high expressions for small RNAs that were shorter than 75 bp (equal to the read length in our study), we randomly selected a few small RNAs and mapped the reads to the corresponding genes in the reference genome. The expression of miRNA 4459 in *Blood_RiboZ* was 29 RPKM, but was undetected in *Blood_PolyA*. MiRNA 4459 is only 66-bp long, and overlaps with the intronic region of *ARL15*. The read coverage profile pattern (Supplementary Fig. S6) indicated that the reads assigned to miRNA 4459 were more likely derived from *ARL15*. Likewise, intronic reads that originated from *KAT6A* were wrongly counted towards *SNORD112* (Supplementary Fig. S7) in *Blood_RiboZ* and *Colon_RiboZ*. *SNORD112* is only 60-bp long, and thus its mature transcript was unlikely to be sequenced even if this gene was truly expressed in the samples. The read coverage pattern in Supplementary Fig. S7 does not corroborate its high expression values in *Blood_RiboZ* and *Colon_RiboZ* at all. Genome wide, 27% of the annotated small RNAs were located in protein-coding or lncRNA genes^35^, and thus the expression levels of these small RNAs tended to be overestimated in the rRNA depletion RNA-seq. Likewise, the gene quantification results for other genes are likely to be exaggerated if their exons overlap with the introns of other genes; for instance, TCR J gene fragments, as will be discussed below. Thus, in general, the estimated gene expression for small RNAs in the rRNA depletion method tends to be less accurate than the polyA+ selection method. This is another important reason why we recommend polyA+ selection rather than rRNA depletion for gene quantification.

TCRs/BCRs are generated by the recombination of V, D, and J gene segments. The TCR J fragments are very short. Like small RNAs, they are quite often overestimated because the majority of TCR J genes overlap with the long intronic region of TCAC (T-cell receptor alpha constant). For example, the estimated expression of *TRAJ12* (T-cell receptor alpha joining 12) in *Blood_RiboZ* was about 130 RPKM, but the read coverage pattern did not corroborate such a high expression level (Supplementary Fig. S8). *TRAJ12* is only 60-bp long, which is much shorter than the read length of 75 bp. This gene is joined with another variable gene through V (D) J recombination to form a TCR molecule. If *TRAJ12* was truly expressed, we should have detected exon–exon junctional reads that spanned *TRAJ12* and its associated variable gene, but no such junctional reads were observed (Supplementary Fig. S8). Human TCR J genes form a cluster in chromosome 14. When the visual region was expanded to include more individual TCR J gene fragments (Supplementary Fig. S9), it became evident that the expression levels of most J genes were overestimated. The counted reads in those TCR J genes were likely to have come from TCAC intronic regions.

### Investigation and analysis of intron rate at the gene level

As shown in Fig. 3, a high fraction of sequences were mapped to intronic regions in the rRNA depletion RNA-seq. To quantify the percentages of intronic read at the gene level, the number of reads mapped to intronic and exonic regions of individual genes were counted separately, and then the intron rate (number of intronic reads divided by the sum of the numbers of intronic and exonic reads) was calculated for each gene. A gene was excluded from the statistical analysis if it overlapped with any other gene, or had no introns, or its expression level was <1 RPKM in any sample. After stringent filtering, 135 lncRNAs and 5823 protein-coding genes survived.

**Figure 6:**
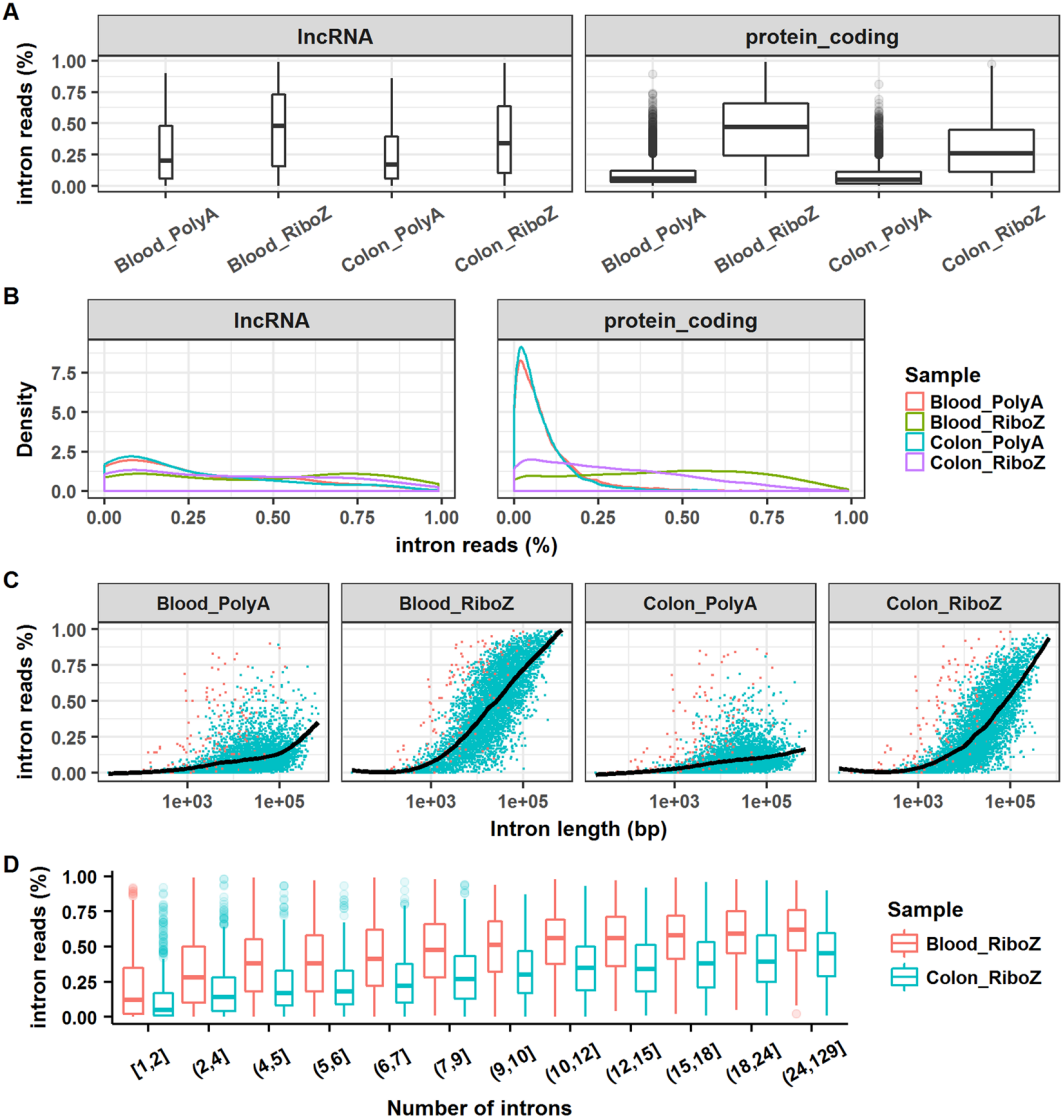
Analyses of intron rate at the gene level. (**A**) Boxplots of intron rate for lncRNA and protein-coding genes. (**B**) Density plots of intron rate for lncRNA and protein-coding genes. (**C**) Relationship between intron rate and intron length. (**D**) Relationship between intron rate and the number of introns in a gene. The intron rate was calculated as follows. First, the number of reads mapped to intronic and exonic regions of individual genes were counted. Then, the intron rate was calculated by dividing the number of intronic reads by the sum of the numbers of intronic and exonic reads.

Boxplots and density plots of intron rates are displayed in Fig. 6A and B. In the *Blood_RiboZ* sample, the median intron rates were close to 0.5 for both lncRNA and protein-coding genes, whereas, in the *Colon_RiboZ* sample, the median intron rates were lower (0.34 and 0.26 for lncRNA and protein-coding genes, respectively). As shown in Fig. 6B, the intron rate distributions for *Blood_PolyA* and *Colon_PolyA* were nearly identical, and the majority of protein-coding genes had very low intron rates in the polyA+ selection RNA-seq. However, the distribution of the intron rates displayed different patterns in *Blood_RiboZ* and *Colon_RiboZ*. The relationship between intron rate and gene structure was investigated. In general, the intron rate increased with intron length or with the number of introns in a gene, but the trend was more apparent in *Blood_RiboZ* and *Colon_RiboZ* than in *Blood_PolyA* and *Colon_PolyA* (Fig. 6C and D). In the rRNA depletion RNA-seq, the longer the intron, the higher the intron rate. The relationship between intron rate and intron length or number was also sample-dependent (Fig. 6D).

## Discussion

For the blood sample, a higher proportion of RNA originated from regions outside known exons in the rRNA depletion RNA-seq (78%) compared with the polyA+ selection RNA-seq (29%). Notably, 50% of all mapped sequence reads were located in introns for the rRNA depletion, whereas only 6% were located in introns for the polyA+ selection (Fig. 2A). The intronic reads either originated from independent transcripts or were immature transcripts that had not been spliced. Immature transcripts could include either full-length pre-mRNA molecules or nascent transcripts where the RNA polymerase had not yet attached to the 3′ end of the gene. Recent RNA-seq studies^36,37^ have suggested that in rRNA depletion RNA-seq, intronic reads come mainly from immature transcripts, predominantly from nuclear RNAs. In the polyA+ selection protocol, the presence of a small portion of intronic reads (~6% in blood) might represent background oligo(dT) priming to stretches of adenines in primary transcripts, rather than true polyadenylated transcripts^36^. Or a fraction of polyA-selected intronic RNA may represent transcripts that undergo splicing after polyadenylation. A previous study also suggested that polyA+ purification was not completely efficient^38^.

The majority of RNA-seq projects that used polyA+ selection have interrogated the polyA+ fraction of RNAs extracted from tissues or blood cells, assuming that most known mature mRNAs are polyadenylated. It has generally been assumed that protein-coding genes have polyA+ tails, while lncRNA do not. However, this view appears to be wrong. Our results show that some lncRNAs and pseudogenes were detected by the polyA+ selection method (Fig. 3D), indicating that non-coding RNAs may also be polyadenylated and, therefore, captured along with mRNAs using oligo (dT) primers. Whether a transcript contains a polyA+ tail may not be determined by its gene biotype, but rather conditionally dependent. Even under the same condition, some gene transcripts can coexist as polyA+ and polyA− populations^29^. Replication-dependent histone mRNAs are known to lack polyA+ tails, but this is not universally true. In our study, *HIST1H2AC* was highly expressed in blood and detected by the polyA+ selection method (Supplementary Fig. S4), and its polyA+ signature was verified independently by 3′-end sequencing (Supplementary Fig. S5). Besides, a subset of histone genes was found to produce polyA+ mRNAs under a variety of cellular conditions^30,31^.

In principle, mRNA quantification through polyA+ selection is considered to be reliable and accurate, and the contribution of nuclear RNA to the total RNA population has been considered negligible for studies focussed on mature coding transcripts^16,36,37^. Obtaining information about the polyA− fraction of the RNAs is the most attractive advantage of using rRNA depletion instead of polyA+ selection. However, rRNA depletion RNA-seq captures many more immature transcripts. Moreover, only mature mRNAs play their biological functions and roles, and should be quantified. Indeed, the immature transcripts captured by rRNA depletion complicate data interpretation in RNA-seq studies. Furthermore, we found the expression for many small RNAs and TCRs/BCRs were overestimated in the rRNA depletion method. Based on these results, polyA+ selection is our recommended approach for RNA-seq projects where the main goal is gene quantification. polyA+ selection RNA-seq is also the procedure of choice for identifying alternative splicing events^16^. However, the RNA-seq data from rRNA depletion provide unique insights into the transcriptional processes in cells^36,39,40^. Ameur *et al*.^36^ analysed the pattern of intronic sequence read coverage and found it agrees very well with co-transcriptional splicing. It is noted that fresh frozen biopsies with high quality RNA are rarely obtained from clinical studies. Many clinical samples are archived FFPE samples, where RNA might have undergone partial degradation. For degraded samples, the rRNA-depleted libraries are better than polyA-selected libraries.

Notably, a few lncRNAs and small RNAs made up a large fraction of sequences. For instance, in the *Blood_RiboZ* sample, 2.68% and 2.69% of the total counted reads were assigned to *MALAT1* and *RN7SL2*, respectively (Fig. 4 and Supplementary Table S1). Such highly expressed genes are problematic because their presence considerably lowers the sequencing depth of the other RNAs. Therefore, we recommend specifically depleting highly abundant lncRNAs and small RNAs in samples by including corresponding specific probes in the rRNA depletion kit, thus improving the sequencing depth of the remaining RNAs.

The distribution of annotated RNAs differs markedly between the cytosolic and nuclear compartments of cells^35^. Coding and non-coding transcripts are localized predominantly in the cytosol and nucleus, respectively. It was found that the small RNA classes were enriched in the cellular compartments where they were known to perform their functions; for example, miRNAs and tRNAs in the cytosol, and snoRNAs in the nucleus^35^. Zaghlool *et al*.^37^ hypothesized that subcellular fractions of RNAs may provide a more accurate picture of gene expression. They demonstrated that RNA-seq of the cytoplasmic fraction produced increased exonic coverage and reduced levels of intronic reads, and that RNA-seq of nuclear RNA was better than RNA-seq of total RNA for measuring nascent transcript levels and for studies of splicing mechanisms. Thus, RNA-seq of cytosolic and nuclear RNAs separately can significantly improve the analysis of mature and nascent transcripts from human blood and tissues.

polyA+ selection and rRNA depletion both selectively omit a distinct set of RNAs, so different fractions of the transcriptome are sequenced; thus, generating incompatible datasets. Aside from rRNAs, polyA+ selection also excludes many other mRNAs lacking polyA+ tails. By contrast, rRNA-depletion can characterize both polyA+ and polyA− RNAs, but also captures nascent transcripts and thus the RNA-seq data contain a large proportion of intronic sequences from pre-mature mRNAs. Despite the overall high concordance between the polyA+ selection and rRNA depletion RNA-seq (Fig. 4), thousands of genes showed large discrepancies between the two methods, which pose a computational challenge for the integration of data from polyA+ selection and rRNA-depleted libraries^41^. To address this problem, Bush *et al*.^41^ developed a systematic means of accounting for library type, which allowed data from these two methods to be compared. This approach could conceivably assist in the novel re-use of existing RNA-seq data.

## Materials and Methods

### Ethics Approval and Consent to Participate

The protocol for the Pfizer Research Support Program to collect blood samples from volunteers was approved by the Schulman Associates Institutional Review Board (IRB#201065670; http://www.sairb.com/Pages/home.aspx). Written informed consent was obtained from all volunteer blood donors for the research described and potential publication thereof. Samples from individuals were coded at the time of collection and then pooled prior to data generation, removing any possible association of analytical measurements with a single donor.

### Blood sample collection, RNA extraction, and globin mRNA and rRNA depletion

Peripheral venous blood samples from five healthy male volunteers were collected in PAXgene Blood RNA tubes (PreAnalytiX GmbH, BD Biosciences, Mississauga, ON, Canada). The blood samples were pooled across subjects to create a single pool. The pooled blood was dispensed into approximately 10-mL aliquots. Total RNA was extracted from four of the aliquots using a PAXgene Blood RNA Kit (cat# 762164, Qiagen, Chatsworth, CA, USA) according to the manufacturer’s protocol. The yield and quality of the isolated RNAs were assessed using a NanoDrop 8000 spectrophotometer (Thermo Fisher Scientific, Waltham, MA, USA) and Agilent 2100 Bioanalyzer (Agilent Technologies, Santa Clara, CA, USA), respectively. The RIN of blood RNA was 7.2. An aliquot of 1.5 μg of each RNA was further processed with a GlobinClear kit (cat# AM1980, Thermo Fisher Scientific) to remove globin mRNA. After globin RNA depletion, the yield and quality of the resulting RNA were assessed again using a NanoDrop 8000 and Agilent 2100 Bioanalyzer. Another aliquot of 1 μg of each RNA was processed using a Globin-Zero Gold rRNA Removal Kit (cat# GZG1206, Illumina, San Diego, CA, USA) to remove rRNA (cytoplasmic and mitochondrial) and any remaining globin mRNA. Then, 1 μl of each resulting RNA was assessed with an Agilent 2100 Bioanalyzer using an RNA 6000 Pico Chip to verify the depletion of rRNA.

### Colon RNA and rRNA depletion

We purchased 100 μg Human Colon Total RNA from Thermo Fisher Scientific (cat# AM7986). The RNA was dispensed into 2 μg aliquots and stored at −80 °C before being analysed. Four aliquots of 1 μg colon RNA were treated with Ribo-Zero Gold rRNA Removal Kit (cat# MRZH126, Illumina) to deplete rRNA (cytoplasmic and mitochondrial). Then, 1 μl of each resulting RNA was assessed with an Agilent 2100 Bioanalyzer using an RNA 6000 Pico Chip to verify the depletion of rRNA.

### cDNA library construction and sequencing

To generate polyA+ selection RNA-seq data, eight cDNA libraries were prepared from 300 ng of each GlobinClear-treated blood RNA sample (n = 4) and 300 ng of each colon RNA sample (n = 4) using a TruSeq Stranded mRNA Library Prep kit (cat# 20020594, Illumina). To generate rRNA depletion RNA-seq data, eight cDNA libraries were prepared from each Globin-Zero-treated blood RNA sample (n = 4) and each Ribo-Zero Gold-treated colon RNA sample (n = 4) using a TruSeq Stranded Total RNA Library Prep kit (cat# 20020596, Illumina). The eight mRNA-seq libraries and eight total RNA-seq libraries were sequenced in two separated runs on a NextSeq. 500 platform (Illumina) using paired-end sequencing (2 × 76 bases). About 50 to 80 million pairs of reads were generated from each library. We used stranded RNA-seq rather than non-stranded RNA-seq because it provides more accurate estimates of transcript expression^42^.

### Reads mapping and counting, and differential expression analysis

Gene quantification results are dependent on the choice of gene annotations^43,44^. Previously, we evaluated the impact of different annotations on RNA-seq data analysis^44^. In this paper, we used the human genome GRCh38 and Gencode Release 25^20^ annotations to map and count sequence reads. The reads were mapped to the GRCh38 reference genome using STAR^45^ v2.5.2 h. The parameters chosen for the STAR run were “*–runThreadN 8;–alignSJDBoverhangMin 1;–outReadsUnmapped Fastx;–outFilterMismatchNoverLmax 0.05;–outFilterScoreMinOverLread 0.90;–outFilterMatchNminOverLread 0.90;–alignIntronMax 1000000;–outSAMtype BAM SortedByCoordinate*”. The union-exon based approach was used for gene quantification^46^, and featureCounts^47^ was used to count reads mapped to individual genes. The parameter used in featureCounts run was *“–minOverlap 25*”. Only the reads that uniquely mapped to exonic regions were counted, and reads that mapped to gene overlapping regions were excluded. Differential expression analysis was performed using the edgeR^21^ package.

### Data availability

All the raw sequencing reads generated in this study have been submitted to the NCBI Sequence Read Archive and are available under the accession number SRP127360.

## Electronic supplementary material

Supplementary Materials
