# Supplementary material for: Evaluation of two main RNA-seq approaches for gene quantification in clinical RNA sequencing: polyA+ selection versus rRNA depletion
Source: Sci Rep. 2018 Mar 19;8:4781. doi: 10.1038/s41598-018-23226-4 (PMC5859127; doi:10.1038/s41598-018-23226-4)
Supplement: Supplementary file 1 — Supplementary Materials [file 41598_2018_23226_MOESM1_ESM.pdf]

Evaluation of two main RNA-seq approaches for gene quantification in clinical RNA sequencing: polyA+ selection versus rRNA depletion

Shanrong Zhao, Ying Zhang, Ramya Gamini, Baohong Zhang, David von Schack

Supplementary Table S1. Genes (labeled in Figure 4) with exceptionally high expressions and large difference between the polyA+ selection and rRNA depletion methods in blood and colon samples

**gene_name**	**category**	**CPM (Count Per Million)**			
		Blood_PolyA	***Blood_RiboZ***	***Colon_PolyA***	***Colon_RiboZ***
MALAT1	lncRNA	246.02	26801.2	248.51	3237.79
NEAT1	lncRNA	385.1	4919.36	1665.09	737.52
HBA2	protein_coding	2388.99	1.79	0.42	0.13
MTATP6P1	pseudogene	550.45	2.34	1356.58	7.66
RN7SL3	smallRNA	1.5	855.58	0.17	96.67
RN7SL2	smallRNA	43.6	26875.88	34.43	18289.55
SNORD3A	smallRNA	0.17	113.29	3.68	1067.02
IGHA1	TCR/BCR	439.46	51.83	11805.19	3172.32
IGHA2	TCR/BCR	95.98	11.19	4338.99	1494.27
IGHM	TCR/BCR	795.99	165.93	1835.51	997.19
IGKC	TCR/BCR	551.1	158.43	8564.36	3974.03

**gene_name**	**category**	**Relative abundance in its own category (%)**			
		Blood_PolyA	***Blood_RiboZ***	***Colon_PolyA***	***Colon_RiboZ***
MALAT1	lncRNA	2.07	47.46	2.32	20.56
NEAT1	lncRNA	3.24	8.71	15.56	4.68
HBA2	protein_coding	0.24	0	0	0
MTATP6P1	pseudogene	12.71	0.03	26.69	0.17
RN7SL3	smallRNA	1.11	2.81	0.13	0.44
RN7SL2	smallRNA	32.32	88.39	26.03	83.95
SNORD3A	smallRNA	0.13	0.37	2.78	4.9
IGHA1	TCR/BCR	9.25	3.46	34.75	23.26
IGHA2	TCR/BCR	2.02	0.75	12.77	10.95
IGHM	TCR/BCR	16.76	11.08	5.4	7.31
IGKC	TCR/BCR	11.6	10.58	25.21	29.13

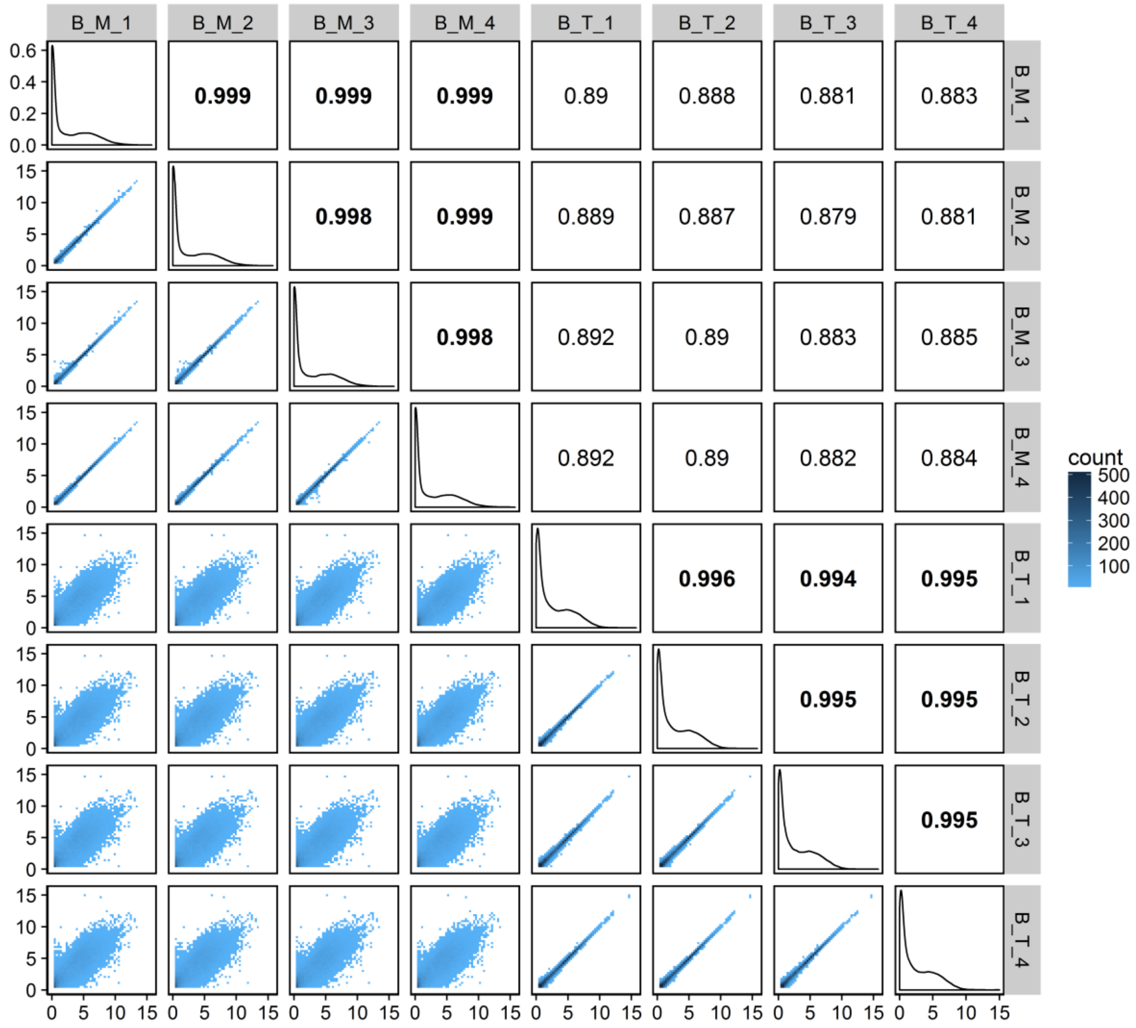

Supplementary Figure S1. The scatter plots and correlations for all blood replicates. The correlations among replicates sequenced by the same protocol are very high, while the correlations between different protocols get worse.

Calculation of correlation: First, lowly expressed genes (RPKM < 0.5 across all samples) were filtered out, and then correlation was calculated using log2(CPM+1). CPM: Count Per Million.

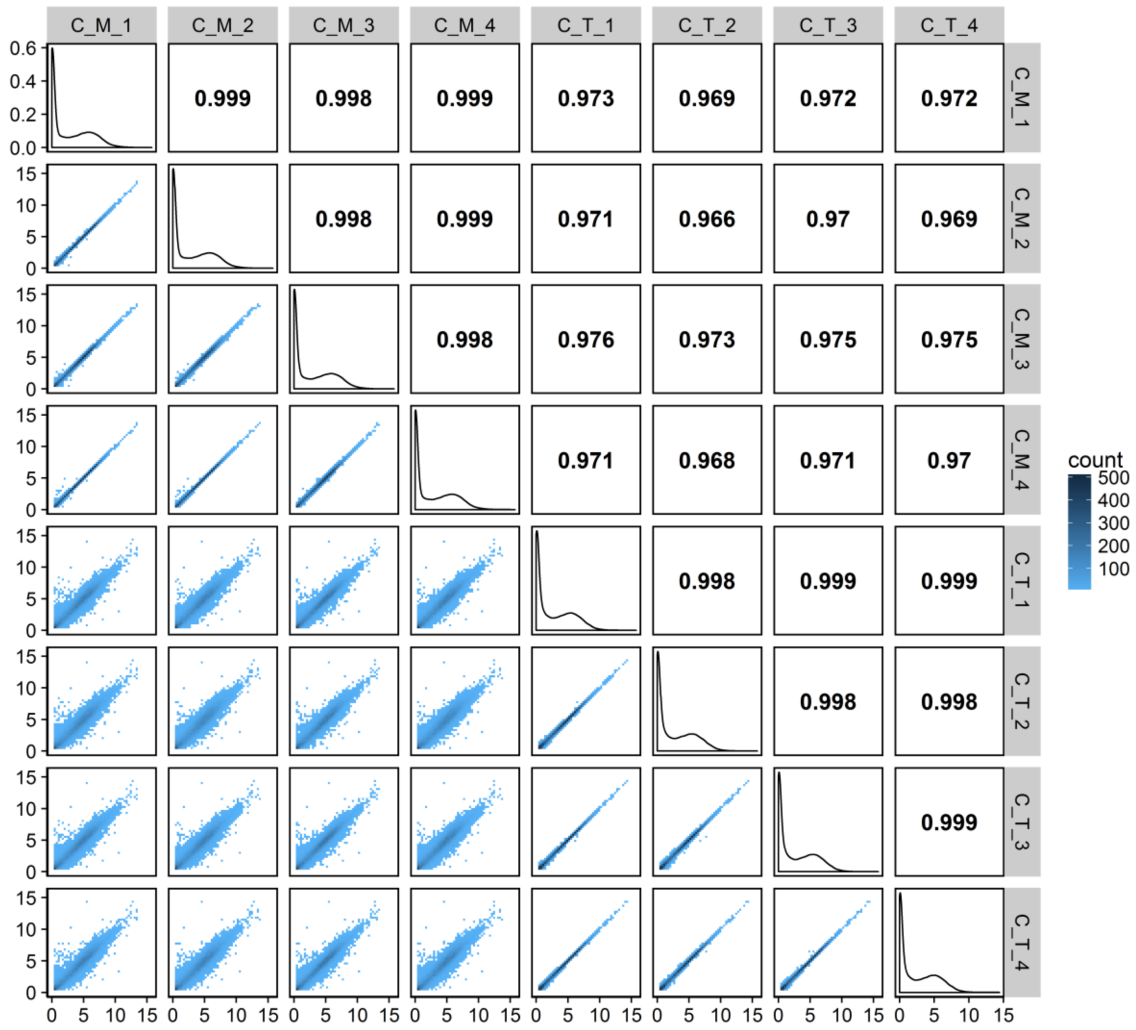

Supplementary Figure S2. The scatter plots and correlations for all colon replicates. The correlations among replicates sequenced by the same protocol are very high, while the correlations between different protocols get worse.
Calculation of correlation: First, lowly expressed genes (RPKM < 0.5 across all samples) were filtered out, and then correlation was calculated using log2(CPM+1). CPM: Count Per Million.

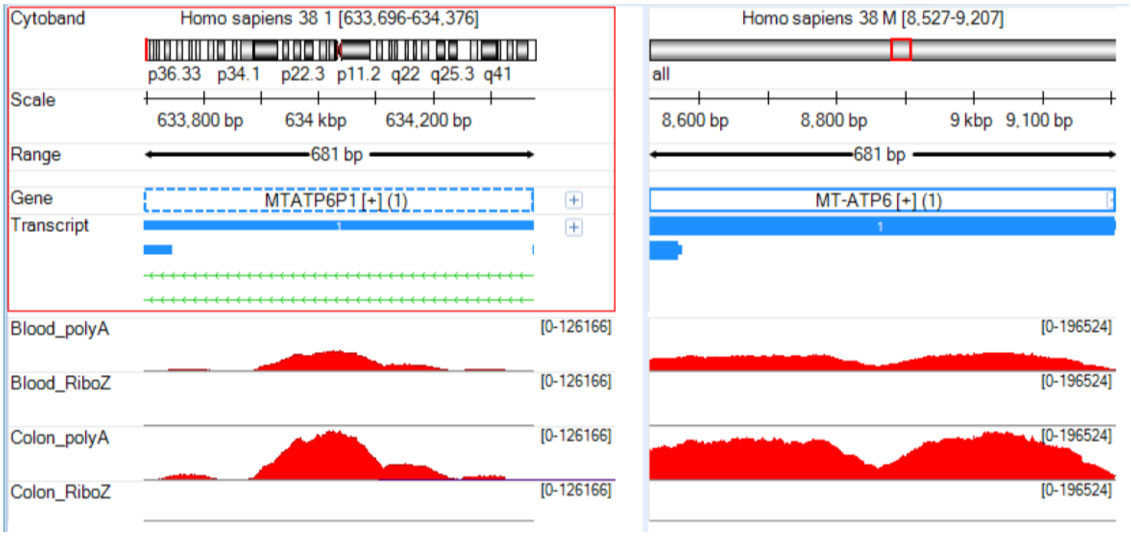

Supplementary Figure S3. MTATP6P1 (mitochondrially encoded ATP synthase 6 pseudogene 1) and MTATP6 are highly expressed in the polyA+ selection RNA-seq in both blood and colon samples, but not in the rRNA depletion method. The sequences of MTATP6P1 and MTATP6 are nearly identical. MTATP6 is a known gene in mitochondria. It is speculated that both MTATP6 and MTATP6P1 transcripts are likely to be depleted by Globin-Zero Gold rRNA Removal Kit.

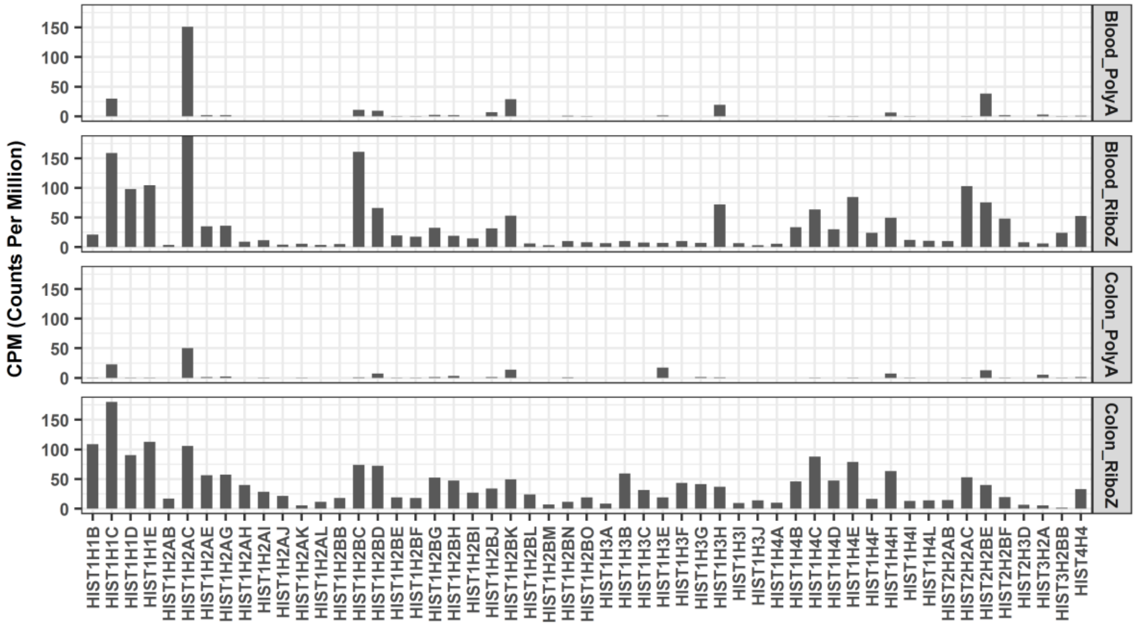

Supplementary Figure S4. The histone genes are known to be expressed as replication-dependent polyA- transcripts, and thus are barely detected in the polyA+ selection RNA-seq. However, HIST1H2AC and a few other histone genes are exceptions.

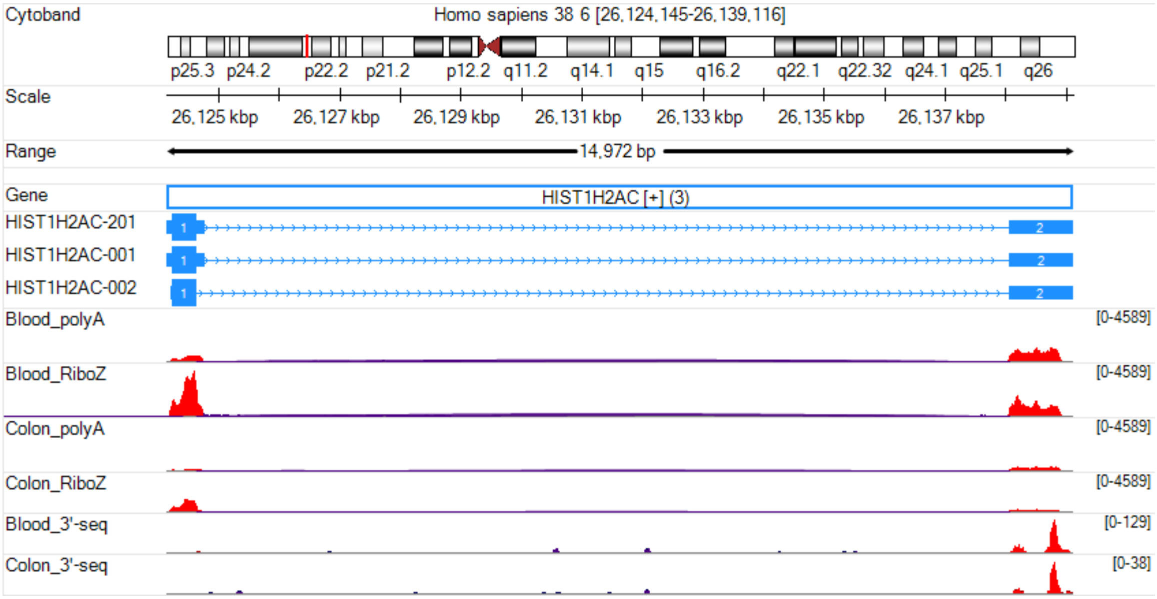

Supplementary Figure S5. The read coverage pattern for HIST1H2AC verifies its high expression. Indeed, the HIST1H2AC transcript has a polyA+ tail, and this is confirmed by 3'-end polyA+ sequencing.

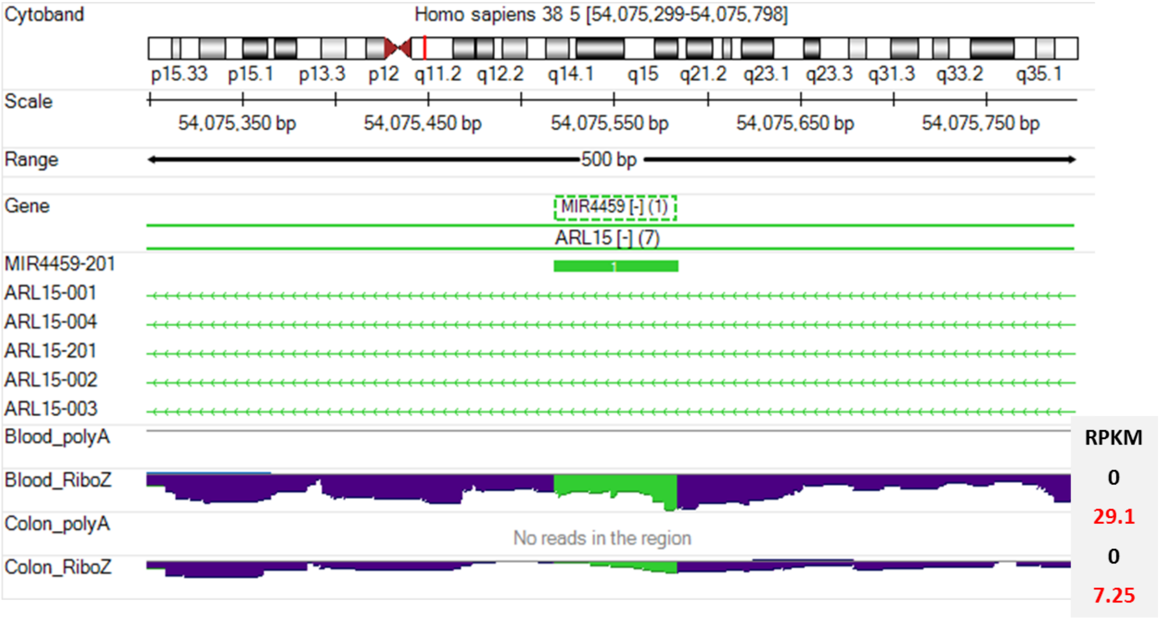

Supplementary Figure S6. MIR4459 is only 66bp long, and it overlaps with the intronic region of the gene ARL15.The reported high expression of MIR4459 in *Blood_RiboZ* is wrong. The read coverage pattern indicates those reads assigned to MIR4459 are actually derived from intron region of ARL15.

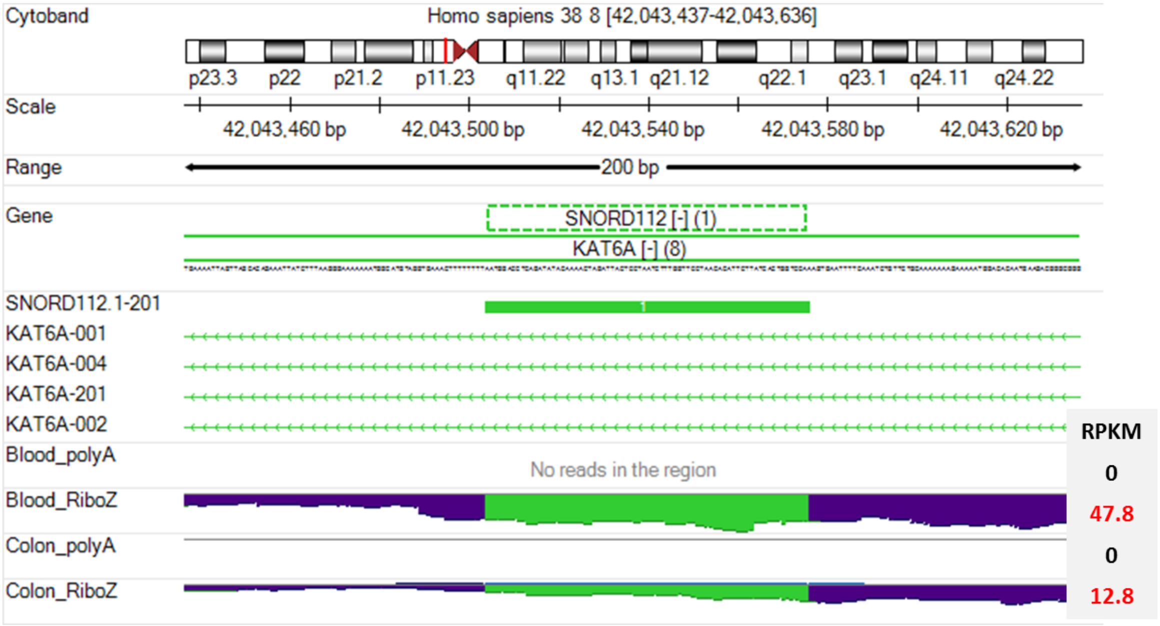

Supplementary Figure S7. Those intronic reads originating from KAT6A are wrongly counted towards SNORD112 in *Blood_RiboZ* and *Colon_RiboZ*. SNORD112 is only 60bp, and presumably removed in the size selection step in RNA-seq library preparation even if this gene is truly expressed. The reads coverage pattern clearly does not corroborate the expression of SNORD112 at all.

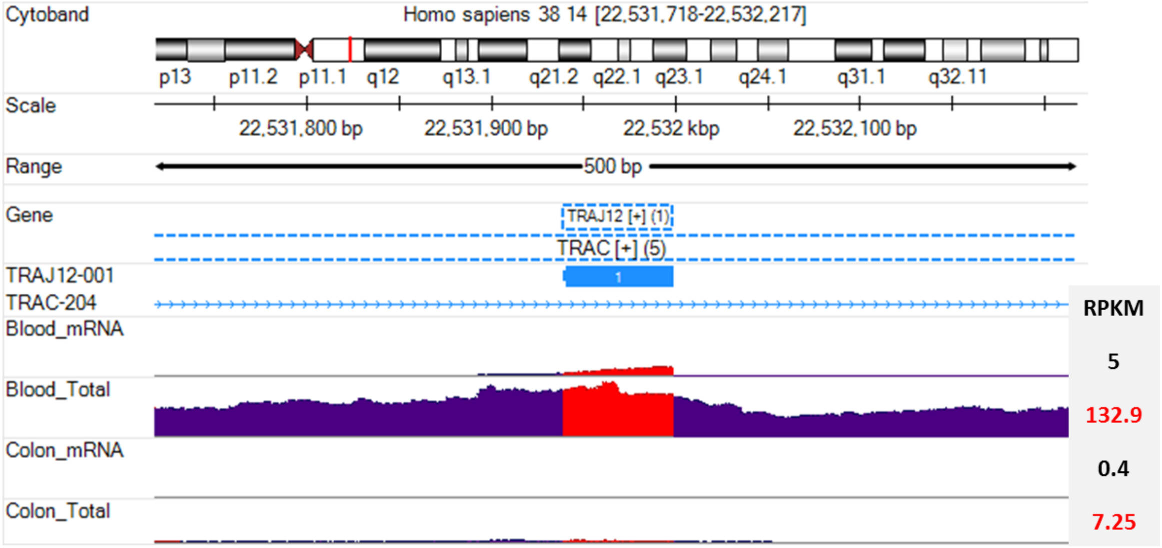

Supplementary Figure S8. TRAJ12 is 60bp long, shorter than the read length of 75bp. TRAJ12 overlaps with an long intron in TRAC (T-Cell Receptor Alpha Constant), and its expression in the *Blood_RiboZ* is overestimated. TRAJ12 is supposed to be joined with another variable gene through V(D)J recombination to form a TCR molecule. If so, exon-exon junctional reads spanning TRAJ12 and its associated variable gene should be seen. Unfortunately, none of such junctional reads are observed.

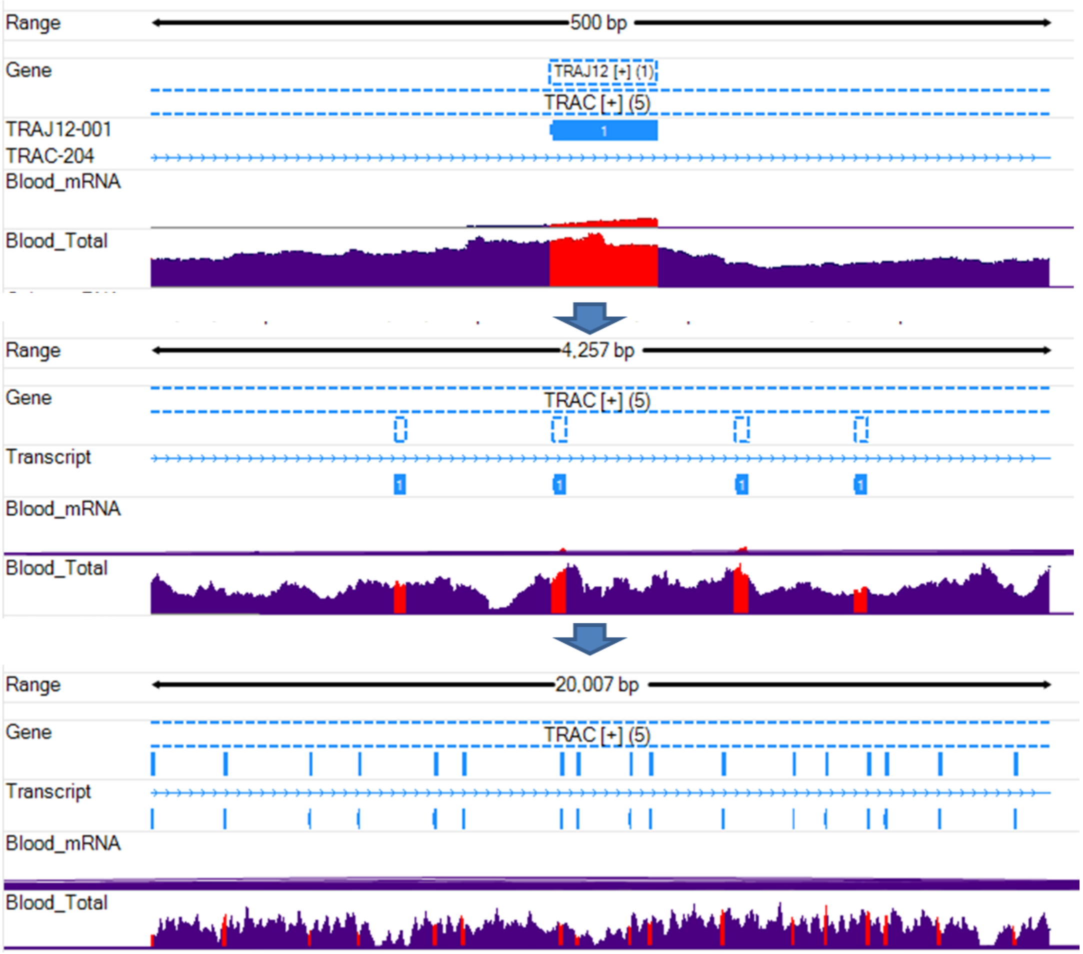

Supplementary Figure S9. Human TCR J genes form a cluster in chromosome 14. As more and more J gene fragments are zoomed into the visible region, it becomes more and more evident that the expression levels for most J genes are overestimated in the rNRA depletion RNA-seq. The read coverage pattern indicates those reads counted to TCR J gene fragments actually originate from the intron region of TCAC.
